# Polyphenolic compounds in combating MDR periodontal pathogens: current research and future directions

**DOI:** 10.3389/fphar.2025.1678979

**Published:** 2025-11-12

**Authors:** Nada Tawfig Hashim, Rasha Babiker, Vivek Padmanabhan, Md Sofiqul Islam, Riham Mohammed, Sivan Padma Priya, Nallan C. S. K. Chaitanya, Shahistha Parveen Dasnadi, Ayman Ahmed, Bakri Gobara Gismalla, Muhammed Mustahsen Rahman

**Affiliations:** 1 Department of Periodontics, RAK College of Dental Sciences, RAK Medical and Health Sciences University, Ras Al-Khaimah, United Arab Emirates; 2 Department of Physiology, RAK College of Medical Sciences, RAK Medical and Health Science University, Ras Al-Khaimah, United Arab Emirates; 3 Department of Pediatric and Preventive Dentistry, RAK College of Dental Sciences, RAK Medical and Health Sciences University, Ras Al-Khaimah, United Arab Emirates; 4 Department of Operative Dentistry, RAK College of Dental Sciences, RAK Medical and Health Sciences University, Ras Al-Khaimah, United Arab Emirates; 5 Department of Oral Surgery, RAK College of Dental Sciences, RAK Medical and Health Sciences University, Ras Al-Khaimah, United Arab Emirates; 6 Department of Oral Pathology, RAK College of Dental Sciences, RAK Medical and Health Sciences University, Ras Al-Khaimah, United Arab Emirates; 7 Department of Oral Medicine and Radiology, RAK College of Dental Sciences, RAK Medical and Health Sciences University, Ras Al-Khaimah, United Arab Emirates; 8 Department of Orthodontics, RAK College of Dental Sciences, RAK Medical and Health Sciences University, Ras Al-Khaimah, United Arab Emirates; 9 Department of Periodontology and Implantology, Faculty of Dentistry, Nile University, Khartoum, Sudan; 10 Department of Oral Rehabilitation, Faculty of Dentistry, University of Khartoum, Khartoum, Sudan

**Keywords:** polyphenols, flavonoids, curcumin, resveratrol, epigallocatechin gallate (EGCG), quercetin, periodontitis, antimicrobial resistance

## Abstract

Multidrug-resistant periodontal pathogens such as Porphyromonas gingivalis, Aggregatibacter actinomycetemcomitans, and *Fusobacterium* nucleatum present growing challenges to conventional antibiotic therapy, driving the search for alternative or adjunctive approaches. Polyphenolic compounds, derived from a wide range of plant sources, have emerged as promising candidates because of their antimicrobial, anti-inflammatory, and host-modulatory properties. A broad review of studies published between 2010 and 2025 highlights the multitargeted mechanisms of key polyphenols, including epigallocatechin-3-gallate, curcumin, resveratrol, and quercetin. These compounds disrupt bacterial membranes, inhibit efflux pumps, downregulate virulence genes, and interfere with quorum-sensing pathways and biofilm maturation, while also attenuating NF-κB signaling and pro-inflammatory cytokines. Several investigations demonstrate synergistic effects with antibiotics, enhancing membrane permeability, biofilm penetration, and dose-sparing efficacy. At the same time, advances in nanotechnology—such as nanoparticles, liposomes, mucoadhesive systems, and smart gels—have begun to overcome the inherent challenges of poor solubility, instability, and short oral residence that limit the therapeutic use of polyphenols. Despite these encouraging developments, variability in extraction methods, lack of standardization, and a scarcity of large, well-designed clinical trials remain significant obstacles to translation. Overall, the accumulating evidence suggests that polyphenols hold strong potential as sustainable adjuncts for managing resistant periodontal infections, offering dual benefits of antimicrobial activity and host modulation. Future progress will depend on harmonizing formulations, refining targeted delivery, and validating outcomes in robust clinical settings.

## Introduction

Periodontitis is a biofilm-mediated, multifactorial inflammatory disease driven by dysbiotic microbial communities and sustained by an abnormal host immune response (Martínez-García and Hernández-Lemus, 2021). It remains a major clinical and public health concern. Antibiotics are used selectively in advanced cases, particularly Stage III and IV, Grade C periodontitis characterized by rapid progression, and high-risk modifiers according to the 2017 classification (Cosgarea et al., 2022). However, the growing emergence of multidrug-resistant (MDR) periodontal pathogens such as Porphyromonas gingivalis (*P.gingivalis*), Aggregatibacter actinomycetemcomitans (*A. actinomycetemcomitans*), and *Fusobacterium* nucleatum (*F. nucleatum*) threatens the long-term efficacy of conventional antibiotic regimens (Rams et al., 2023). This rising resistance underscores the urgent need for adjunctive treatment strategies that not only reduce microbial viability but also disrupt biofilm architecture and modulate host immunity while minimizing adverse effects (Zhao et al., 2023).

Natural polyphenolic compounds, present in a wide range of plant-derived foods and traditional herbal medicines, are gaining recognition for their antimicrobial and host-modulatory potential in periodontal therapy (Hashim et al., 2024). Their diverse bioactivities include inhibition of bacterial adhesion, suppression of quorum sensing, disruption of membrane integrity, interference with virulence factors such as proteases and hemolysins, and modulation of inflammatory signaling pathways (Shariati et al., 2024). Compounds such as quercetin, curcumin, catechins, and resveratrol demonstrate potent antimicrobial and anti-inflammatory effects *in vitro* and *in vivo*, opening new therapeutic opportunities for periodontal treatment (Inchingolo et al., 2022).

Despite this growing body of evidence, clinical translation of polyphenols into mainstream periodontal care remains limited (Hashim et al., 2024; Basu et al., 2018). Several obstacles persist along the bench-to-chairside pipeline. Most available studies are confined to planktonic bacterial cultures, which fail to capture the complexity of multispecies biofilms *in vivo* (Zhao et al., 2023; Hashim et al., 2025a). Furthermore, a lack of standardized protocols for polyphenol extraction, purification, and dosing contributes to variability in reported antimicrobial efficacy (Brglez et al., 2016). Polyphenols also are hindered from inherently poor solubility, extensive metabolic transformation, and rapid systemic clearance, necessitating the development of advanced delivery systems such as nanocarriers and mucoadhesive formulations to achieve therapeutic concentrations at target sites (D'Archivio et al., 2010). Moreover, while the actions of individual compounds are well documented, the synergistic or antagonistic interactions of polyphenol mixtures, or their combinations with conventional antimicrobials, remain poorly characterized (Aatif, 2023).

The significance of overcoming these barriers is amplified by the broader systemic implications of periodontitis. Mounting evidence links the disease to cardiovascular disorders, diabetes mellitus, adverse pregnancy outcomes, and respiratory conditions, elevating periodontitis from a localized oral infection to a global public health issue (Nazir, 2017; Kim and Amar, 2006). Microbial dysbiosis and the chronic inflammatory burden not only exacerbate systemic inflammation but also diminish oral health-related quality of life (OHRQoL), particularly in underserved populations (Kamer et al., 2024; Vivek et al., 2021). According to the Global Burden of Disease Study, severe periodontitis ranks among the leading causes of years lived with disability, reflecting its profound socioeconomic impact (Fu et al., 2025; Hashim et al., 2025b). Yet, despite the continued reliance on systemic antibiotics in severe cases, their effectiveness is increasingly undermined by the proliferation of MDR microorganisms within oral biofilms (Ilyes et al., 2024; Mirghani et al., 2022). This trend not only heightens the risk of treatment failure but also contributes to escalating healthcare costs and fuels the global antimicrobial resistance (AMR) crisis (Salam et al., 2022).

Against this backdrop, the exploration of polyphenols as safe, multi-targeted, and biocompatible therapeutic agents is both timely and imperative. By simultaneously addressing microbial resistance, biofilm resilience, and host inflammatory pathways, polyphenols offer a sustainable strategy for integrative periodontal disease management. Therefore, the aim of this review is to synthesize the most up-to-date and mechanistically grounded evidence on polyphenolic compounds against MDR periodontal pathogens, with particular focus on advanced *in vitro* biofilm models, strategies to overcome bioavailability challenges, and novel formulation approaches that enhance their translational potential.

## Methods (narrative search approach)

Although this work is a narrative review rather than a systematic review, we adopted a structured search strategy to ensure a comprehensive overview of the literature. Electronic databases including PubMed, Scopus, Web of Science, and Google Scholar were searched for articles published between January 2010 and June 2025. The search combined terms such as *polyphenols*, *flavonoids*, *curcumin*, *resveratrol*, *epigallocatechin gallate (EGCG)*, *quercetin*, *periodontitis*, *antimicrobial resistance*, *biofilm*, *quorum sensing*, and *nanodelivery*.

Inclusion criteria focused on *in vitro* and *in vivo* studies examining the antimicrobial, anti-biofilm, quorum-sensing, or host-modulatory properties of polyphenolic compounds against periodontal pathogens, particularly multidrug-resistant strains. Reviews, meta-analyses, and clinical reports were also considered to provide broader translational context. Articles not in English, unrelated to periodontal pathogens, or lacking primary data were excluded.

### Narrative flow of study selection

**FIGURE 1:**
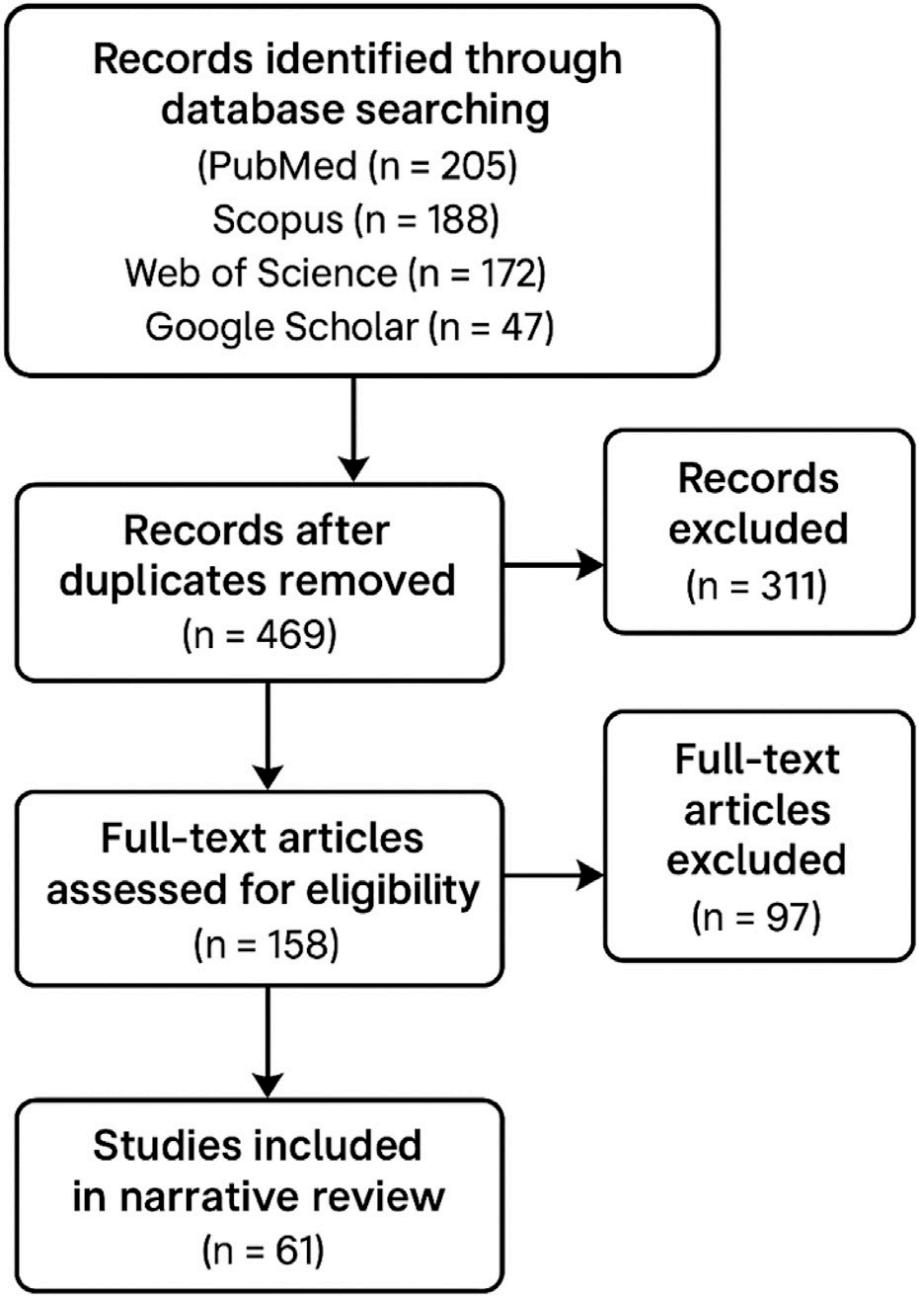
Narrative search flow.

A total of 612 records were identified through database searching (PubMed = 205, Scopus = 188, Web of Science = 172, Google Scholar = 47). After removal of duplicates (n = 143), 469 articles remained for screening. Following title and abstract screening, 311 articles were excluded as irrelevant. The full text of 158 articles was examined, and 97 were excluded due to insufficient mechanistic focus, lack of relevance to multidrug-resistant strains, or non-English language. In total, 61 studies were included in this review (Figure 1).

## Mechanisms of multidrug resistance in periodontal microbiota

Recently, there has been a rapid rise in multidrug-resistant (MDR) strains among major periodontal pathogens—including *P. gingivalis*, Tannerella forsythia (*T. forsythia*), *A. actinomycetemcomitans*, and *F. nucleatum*, posing serious challenges to the efficacy of conventional therapeutic strategies (Łasica et al., 2024). MDR refers to the capacity of microorganisms to withstand multiple classes of antibiotics that were previously effective (Nikaido, 2009). This phenomenon is not solely driven by genetic mutations but is strongly linked to adaptive responses to environmental stress and the ability of bacteria to persist within organized microbial communities such as biofilms (Zhang et al., 2019). The primary resistance driver for periodontal pathogens is their ability to develop and maintain biofilms. These are bacterial cells embedded within a self-constructed extracellular polymeric substance (EPS), which provides a significant survival advantage, as the physical and chemical barrier it presents restricts access to antibiotics (Almatroudi, 2025; Bowen et al., 2017). Within the biofilm, gradients of nutrients, pH, and oxygen establish microenvironments that create a reduced metabolic state within some bacterial populations, rendering them less susceptible to antimicrobial compounds that specifically target cell division during the active phase (Koo et al., 2017). Furthermore, biofilms enhance the development of persister cells, phenotypic subpopulations that exhibit short-term antibiotic resistance despite lacking genes for resistance (Kunnath et al., 2024). These persisters can recolonize the biofilm when antimicrobial pressure decreases, continuing to drive the recurrence of infections (Kunnath et al., 2024). Additionally, the tightly packed architecture of the biofilm promotes horizontal gene transfer between bacteria and facilitates the rapid spread of resistance genes throughout the periodontal niche (Michaelis and Grohmann, 2023).

Other than biofilm-associated tolerance, MDR periodontal pathogens also utilize active efflux mechanisms, such as efflux pumps, to export antibiotics from their intracellular environments (Soto, 2013). These transport proteins cross the cell membrane and can recognize and remove a broad spectrum of antibiotics, thus significantly reducing their intracellular concentrations. Some of the most common include the Resistance-Nodulation-Division (RND) family of pumps found in Gram-negative anaerobes like *P. gingivalis* and the Major Facilitator Superfamily (MFS), which has been implicated in tetracycline resistance among many oral bacteria (Gaurav et al., 2023). ATP-binding cassette (ABC) transporters also operate by using the energy from ATP hydrolysis to power the efflux of macrolides and other antimicrobials. These efflux mechanisms are frequently upregulated during antibiotic stress and lead to cross-resistance, complicating treatment regimens further (Iannelli et al., 2018).

Yet another important resistance mechanism is the enzyme inactivation of antibiotics. Various periodontal pathogens carry the genes for β-lactamases, which are enzymes hydrolyzing the β-lactam ring to inactivate penicillins and cephalosporins (Kakoullis et al., 2021). In addition, aminoglycoside-modifying enzymes phosphorylate, acetylate, or adenylate the antibiotics and thereby render them virtually completely inactive against microorganisms (Egorov et al., 2018). In anaerobic organisms commonly found within periodontal pockets, nitroreductases render metronidazole resistant by changing the active moiety of the drug so that it becomes inert against DNA synthesis (Alauzet et al., 2019). Such resistance enzymes typically occur on plasmids and may be transferred from one bacterium to another very easily and so represent a mirror of how the mouth represents an active pool of resistance elements (Carattoli, 2013).

Modifications in the antibiotic target sites constitute yet another form of resistance that is increasingly recognized in periodontal pathogens. Inhibitory mutations of genes for ribosomal RNA, particularly in the 23S rRNA component, can preclude binding by macrolides and lincosamides (Galgano et al., 2025). DNA gyrase and topoisomerase IV, the target enzymes of fluoroquinolones, are also altered, leading to resistance by diminishing the efficacy of the drugs (Collins and Osheroff, 2024). Changes in penicillin-binding proteins (PBPs), particularly in *A. actinomycetemcomitans* and Prevotella spp., minimize the binding capacity of β-lactam antibiotics. These changes usually happen due to spontaneous mutation and are sustained under selective pressure caused by intermittent or sub-therapeutic application of antibiotics, which is not uncommon in dental therapy (Nauta et al., 2021).

Horizontal gene transfer (HGT) plays a key role in the dissemination of resistance in periodontal microbiota (Roberts and Kreth, 2014). The oral environment of high microbial density and continuous environmental perturbations is conducive to efficient gene transfer by conjugation, transformation, and, to some degree, transduction. Conjugative plasmids, transposons, and integrons are all types of mobile genetic elements, which promote the transfer of the resistance genes between nonpathogenic bacteria and pathogenic bacteria. This interaction allows for multidrug resistance even among harmless oral strains, which are reservoirs for more virulent forms (Tokuda and Shintani, 2024; Toucho et al., 2017).

In addition, periodontal pathogens have vast adaptive plasticity when under stress in their habitat, another complicating factor. Bacteria have the ability to alter their DNA when under hostile conditions such as nutrient unavailability, exposure to toxins, or stress, to become more resistant to treatment (Grevanny et al., 2024). Activation of global regulatory networks, including stringent response and SOS response pathways, enables survival mechanisms such as metabolic process downregulation and upregulation of efflux pumps and DNA repair enzymes (Dawan and Ahn, 2022). Two-component regulatory systems (TCRS) help bacteria cope with changes in their environment by controlling gene expression to maximize their chances of survival from antimicrobial agent attack (Tierney and Rather, 2019).

Community-level resistance also leads to treatment failure in periodontal infection. Within the biofilm, resistant bacteria can degrade or inactivate antibiotics, creating a niche that is extended to neighboring susceptible species—a phenomenon known as the bystander effect (Morley et al., 2019). Moreover, the harmonious metabolic interactions among species in polymicrobial communities enable functional compensation, with loss of antibiotic susceptibility in one strain being compensated by resistance mechanisms in another. This cumulative resistance reduces the reliability of clinical outcomes from routine susceptibility testing and complicates antibiotic selection (Perry et al., 2022).

Clinically, the implications of MDR in periodontal pathogens are profound. Failure of adjunctive antibiotic therapy, persistent or recurrent infections after mechanical debridement, and the potential systemic spread of resistant organisms pose significant threats to both oral and general health (Abe et al., 2022). In particular, the immunocompromised and those with underlying systemic illness such as diabetes or cardiovascular disease are at greater risk. The complexity of periodontal pathogens’ resistance mechanisms necessitates a shift in paradigm from reliance on traditional antibiotics to the creation of novel therapeutic drugs (Abdullah et al., 2024). Natural compounds like polyphenols that possess the ability to inhibit biofilm development, influence efflux pumps, and modulate virulence gene expression are new promising agents to contribute to fighting against multi-drug resistance infections (Hashim et al., 2025b). Further investigation into these alternatives is a requirement to restore treatment efficacy and protect from mounting antimicrobial resistance threats in periodontal therapy.

### Overview of polyphenolic compounds: sources and structures

Polyphenolic compounds represent a diverse group of secondary metabolites found frequently in the plant kingdom (Zagoskina et al., 2023). They are characterized by having greater than one phenol structural unit, which endows them with very potent antioxidant, anti-inflammatory, and antimicrobial properties (Zagoskina et al., 2023). The bioactive compounds have generated a lot of interest in biomedical sciences due to their vast array of biological activities, especially their current use in combating MDR microorganisms, e.g., periodontal pathogens (Hashim et al., 2025a). The significance of polyphenols in periodontal treatment is not only because they are antimicrobial but also because they have the ability to modulate host immune response and disrupt microbial virulence factors such as quorum sensing and biofilm formation (Hashim et al., 2025a).

Structurally, polyphenols are divided by the phenol ring count and structural features that link the rings together. The prominent groups include flavonoids, phenolic acids, stilbenes, lignans, and tannins (Pandey and Rizvi, 2009). Among these, flavonoids form the largest and most well-characterized group. Flavonoids share a common structure with two phenyl rings (A and B) connected by a three-carbon chain that is linked to a heterocyclic ring (C). Based on variations in the degree of hydroxylation, methylation, glycosylation, and oxidation of the C-ring, flavonoids are further classified into flavonols (e.g., quercetin), flavones (e.g., apigenin), flavanones (e.g., naringenin), flavan-3-ols (e.g., catechins), anthocyanidins (e.g., cyanidin), and isoflavones (e.g., genistein) (Šamec et al., 2021). These structural variations have significant effects on their solubility, bioavailability, and biological activity (Dias et al., 2021).

Phenolic acids represent another major class, which are structurally less complex and more differentiated into hydroxybenzoic acids (e.g., gallic acid) and hydroxycinnamic acids (e.g., ferulic and caffeic acid) (Kumar and Goel, 2019). They are mostly found as free or esterified in fruits, vegetables, whole grain foods, and beverages like coffee and wine. Their antimicrobial activity largely depends on their ability to disrupt microbial cell walls, chelate metal ions involved in enzymatic processes, and modulate oxidative stress pathways (Kumar and Goel, 2019).

Stilbenes, e.g., resveratrol, are less common but have also shown considerable antimicrobial and anti-biofilm activity. Resveratrol, which is found predominantly in grapes and red wine, consists of two aromatic rings held together by a two-carbon ethylene bridge and can insert into lipid bilayers and disorganize bacterial membranes (Salehi et al., 2018).

Lignans, which occur in seeds like flaxseed, are made up of two phenylpropane groups joined together with a central carbon-carbon link. While less extensively studied as far as oral pathogens are concerned, they have antioxidant and antimicrobial properties of relevance to the application in oral health (Sangiorgio et al., 2023).

Tannins are high-molecular-weight polyphenols and can also be categorized as condensed tannins (proanthocyanidins) and hydrolyzable tannins (from gallic or ellagic acid). Tannins are typically antibacterial through protein precipitation, enzyme inhibition, and adhesion inhibition in microbes and, therefore, are potential periodontal biofilm drugs (Adamczyk et al., 2017).

The principal dietary sources of polyphenols are fruits (berries, grapes, apples, and pomegranates), vegetables (onions, spinach, and artichokes), legumes, cereals, tea (green and black), red wine, olive oil, coffee, cocoa, and spices (turmeric and cloves). The concentration of these substances in these foods is quite variable with species, growing conditions, and processing. For example, green tea contains particularly high levels of catechins like epigallocatechin-3-gallate (EGCG), while red wine and grapes contain resveratrol and anthocyanins (Rudrapal et al., 2022).

Of special interest, the polyphenolic profile and composition of plant extracts are significantly regulated by the used extraction methods. The yield and stability of isolated compounds are influenced by solvent polarity, temperature, and pH conditions. Progress in the last few years in extraction techniques like supercritical fluid extraction, microwave-assisted extraction, and nanoencapsulation has improved the recovery and delivery of polyphenols for pharmaceutical purposes (Alara et al., 2021).

Pharmacologically, the antimicrobial activity of polyphenols is diverse. They may interfere with bacterial cell membranes, chelate metal ions essential for bacterial growth, inhibit nucleic acid synthesis, and interfere with protein function. Most polyphenols also regulate bacterial communication via quorum sensing pathways, thereby decreasing virulence without considerable selective pressure that normally leads to resistance (Mandal and Domb, 2024). Such a non-lethal mechanism of antimicrobial activity is particularly preferred in the instance of chronic polymicrobial infections like periodontitis (De Rossi et al., 2025).

Polyphenolic compounds constitute a structurally and functionally diverse group of natural products of immense therapeutic potential. Presence in everyday dietetic sources coupled with their nonspecific bioactivity makes them outstanding alternatives or supplements to traditional antibiotics. As resistance to synthetic antimicrobials grows, the pharmacognostic exploration of polyphenols as periodontal drugs is a stimulating and renewable option (Rathod et al., 2023).

### Antibacterial mechanisms of polyphenols against periodontal pathogens

Polyphenolic compounds exert a multifaceted antibacterial effect against periodontal pathogens, making them particularly promising as adjuncts or alternatives to conventional antimicrobials in periodontal therapy (Hashim et al., 2024). Unlike traditional antibiotics that often target a single cellular process and thus foster resistance development, polyphenols interact with multiple microbial targets simultaneously. This multitargeted approach not only limits the emergence of resistance but also makes polyphenols effective in polymicrobial environments such as the periodontal pocket (De Rossi et al., 2025).

One of the primary antibacterial mechanisms of polyphenols is their ability to disrupt the bacterial cell membrane (Hashim et al., 2024). Due to their amphiphilic nature, many polyphenols, such as catechins and flavonoids, can insert into lipid bilayers, increasing membrane permeability (Hashim et al., 2024). This leads to leakage of intracellular contents, collapse of the proton motive force, and ultimately bacterial cell death (Hashim et al., 2025a). Epigallocatechin-3-gallate (EGCG), a major catechin in green tea, has been shown to induce membrane disruption in Porphyromonas gingivalis and *Fusobacterium* nucleatum, two keystone periodontal pathogens. This membrane-targeting effect is particularly advantageous in anaerobic environments where antibiotic penetration is limited (Kong et al., 2022).

In addition to membrane disruption, polyphenols can interfere with microbial enzyme systems and protein synthesis (De Rossi et al., 2025). For instance, gallic acid and quercetin inhibit critical enzymes involved in DNA gyrase and topoisomerase activity, impairing bacterial DNA replication (Alfonso et al., 2022). Other polyphenols such as resveratrol and apigenin have been shown to bind to bacterial ribosomal subunits, thereby hindering protein synthesis and bacterial growth. The broad-spectrum enzyme inhibitory capacity of polyphenols extends to metabolic enzymes, including those involved in glycolysis and oxidative phosphorylation, further impairing bacterial viability (Sun et al., 2024).

Polyphenols also exhibit potent anti-biofilm activity, which is of particular importance in the context of periodontal disease (Basu et al., 2018). Biofilm formation is a key virulence strategy of periodontal pathogens, conferring resistance to both host defenses and antimicrobial agents (Sharma et al., 2023). Polyphenols such as cranberry proanthocyanidins, EGCG, and curcumin have demonstrated the ability to inhibit biofilm formation by reducing bacterial adhesion, interfering with the production of extracellular polymeric substances (EPS), and disrupting pre-formed biofilms. This action not only reduces bacterial load but also enhances the penetration and efficacy of other antimicrobial agents (Ulrey et al., 2014).

A unique mechanism by which polyphenols exert antibacterial effects is through the inhibition of quorum sensing (QS)—the cell-to-cell communication system that regulates virulence gene expression, biofilm maturation, and resistance traits in bacteria (Naga et al., 2023). Several studies have documented that flavonoids like naringenin and kaempferol can suppress the expression of QS regulatory genes such as luxS, thereby attenuating the virulence of *A. actinomycetemcomitans* and *P. gingivalis*. By targeting bacterial communication rather than viability, polyphenols reduce pathogenicity without inducing strong selective pressure, making them a valuable strategy in managing chronic periodontal infections (Juszczuk-Kubiak, 2024; Geoghegan et al., 2009; Wright and Ramachandra, 2022).

Additionally, polyphenols exert metal-chelating activity, sequestering essential metal ions such as iron, zinc, and magnesium that are required for bacterial enzyme activity and growth (Lakey-Beitia et al., 2021). Tannins and gallic acid, in particular, are effective iron chelators. By depriving bacteria of essential cofactors, polyphenols induce metabolic stress and impair growth. This chelating effect is synergistic with their antioxidant properties, as it disrupts redox homeostasis and enhances oxidative damage in microbial cells (Scarano et al., 2023).

The anti-inflammatory and immunomodulatory effects of polyphenols also indirectly contribute to their antibacterial action. In periodontal tissues, chronic inflammation facilitates microbial colonization and tissue destruction. Polyphenols such as curcumin and resveratrol modulate host inflammatory pathways, notably by inhibiting NF-κB activation and downregulating pro-inflammatory cytokines like IL-1β, IL-6, and TNF-α. This creates a less hospitable environment for pathogenic bacteria and supports host tissue healing. Some polyphenols have also been shown to promote the expression of antimicrobial peptides (AMPs) in gingival epithelial cells, further enhancing innate defense (Hashim et al., 2025a).

Finally, the ability of polyphenols to act synergistically with conventional antibiotics adds a valuable dimension to their antimicrobial profile. Many studies have demonstrated that combining polyphenols with antibiotics such as metronidazole, doxycycline, or amoxicillin can enhance antimicrobial efficacy, lower the required dose of antibiotics, and overcome resistance. This synergistic interaction is often attributed to increased membrane permeability, efflux pump inhibition, or interference with bacterial stress response pathways (Manso et al., 2021).

**FIGURE 2:**
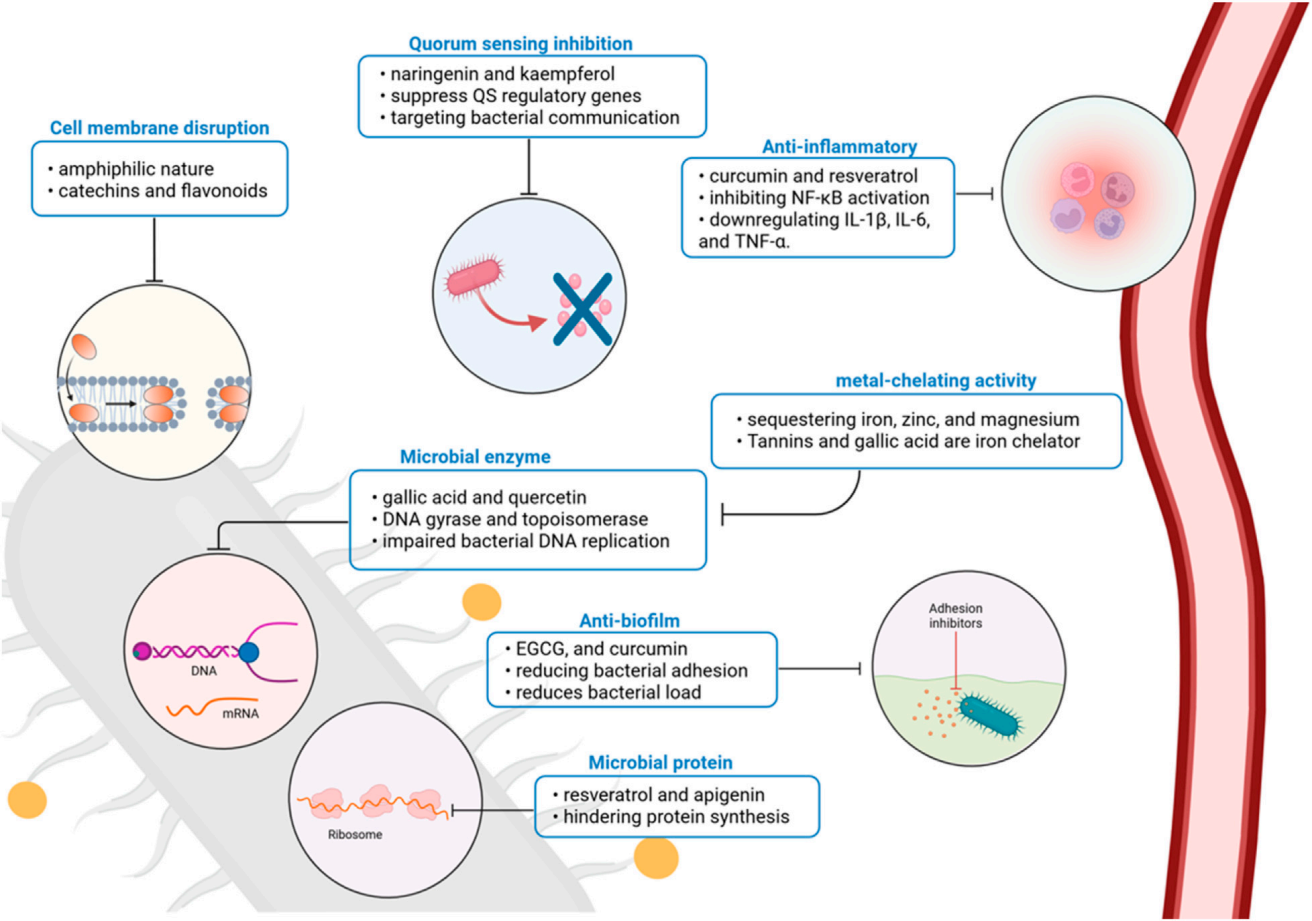
Polyphenolic compounds act through multiple antimicrobial and host-modulatory mechanisms in periodontal disease. Their effects include membrane disruption, enzyme and protein synthesis inhibition, biofilm and quorum-sensing interference, metal chelation, and suppression of pro-inflammatory signaling. Together, these actions reduce bacterial virulence and support host immune modulation, positioning polyphenols as promising adjuncts in periodontal therapy. Created with Biorender.com.

The antibacterial mechanisms of polyphenols against periodontal pathogens are diverse and multifactorial, targeting membrane integrity, biofilm architecture, protein synthesis, quorum sensing, and host immunity (De Rossi et al., 2025). Their ability to interfere with multiple stages of bacterial pathogenesis without promoting resistance makes them attractive candidates for integration into modern periodontal therapy. Future research should focus on optimizing their delivery, improving their bioavailability, and validating their efficacy in clinical settings through well-designed trials (Zhao et al., 2020) (Figure 2).

### Synergistic effects of polyphenols with conventional antibiotics

The increasing prevalence of MDR periodontal pathogens has necessitated the search for combination therapies that enhance antimicrobial action with a low risk of resistance emergence (Bharadwaj et al., 2022). In this context, the synergistic interaction between polyphenolic compounds and conventional antibiotics is an appealing approach. Polyphenols, due to their diverse modes of action, possess the ability to enhance antibiotic activity through multiple avenues such as membrane disruption, efflux pump inhibition, interference with the expression of resistance genes, and enhanced penetration of antibiotics into biofilms. This synergy, not only will lead to enhanced bacterial elimination but also possibly allow for lower doses of antibiotics, thus lessening systemic toxicity and risk of resistance emergence (De Rossi et al., 2025; Liu et al., 2025).

Mechanistically, polyphenols such as epigallocatechin-3-gallate (EGCG), quercetin, curcumin, and resveratrol have been shown to destabilize bacterial cell membranes, rendering them more permeable. This allows antibiotics such as metronidazole, amoxicillin, and doxycycline to enter pathogenic bacteria such as *P. gingivalis*, *A. actinomycetemcomitans*, and *T. forsythia* more effectively (Cione et al., 2019). For example, EGCG has been shown to synergize with β-lactam antibiotics by breaking down the outer membrane of Gram-negative bacteria, which boosts the killing power of amoxicillin. Curcumin has also been shown to improve the effectiveness of tetracyclines by decreasing the activity that pumps the drugs out of the bacteria and decreasing the levels of resistance genes (Chawla et al., 2022).

Polyphenols also exhibit efflux pump inhibition, a key mechanism through which MDR bacteria efflux antibiotics. Through the disruption of the function or expression of efflux transporters such as Resistance-Nodulation-Division (RND) and Major Facilitator Superfamily (MFS) families, polyphenols increase the intracellular levels of antibiotics and restore bacterial susceptibility (Sharma et al., 2023). This action has been demonstrated *in vitro* where catechins synergized fluoroquinolone activity against *F. nucleatum* and Prevotella species even in the presence of intrinsic resistance in the strains (Okuda et al., 2012; Higuchi et al., 2024).

The other significant function of polyphenols in antibiotic synergism is their anti-biofilm action (Slobodníková et al., 2016). In periodontal disease, biofilms protect bacterial populations from the action of antibiotics (Li et al., 2023). Polyphenolic compounds such as proanthocyanidins and ellagic acid interfere with bacterial adhesion, extracellular matrix production, and quorum sensing, destabilizing the biofilm structure. With synergy with antibiotics, this interference facilitates enhanced penetration of antibiotics and enhanced bactericidal activity (Fydrych et al., 2025). For example, the combination of cranberry proanthocyanidins with metronidazole showed superior biofilm eradication in mixed-species subgingival models compared to either agent alone (Bueno-Silva et al., 2023).

Besides direct microbiological effect, polyphenols may also regulate host response, contributing to the therapeutic effect when given in conjunction with antibiotics. Through downregulation of pro-inflammatory cytokines and reduction of oxidative stress on periodontal tissues, they enable healing of the tissues and improve the overall microenvironment, rendering it less conducive to survival of the pathogen. This adjunctive effect could be particularly useful in chronic periodontal infections, where ongoing inflammation maintains dysbiosis and resistance formation (Guo et al., 2022; Jayusman et al., 2022).

**FIGURE 3:**
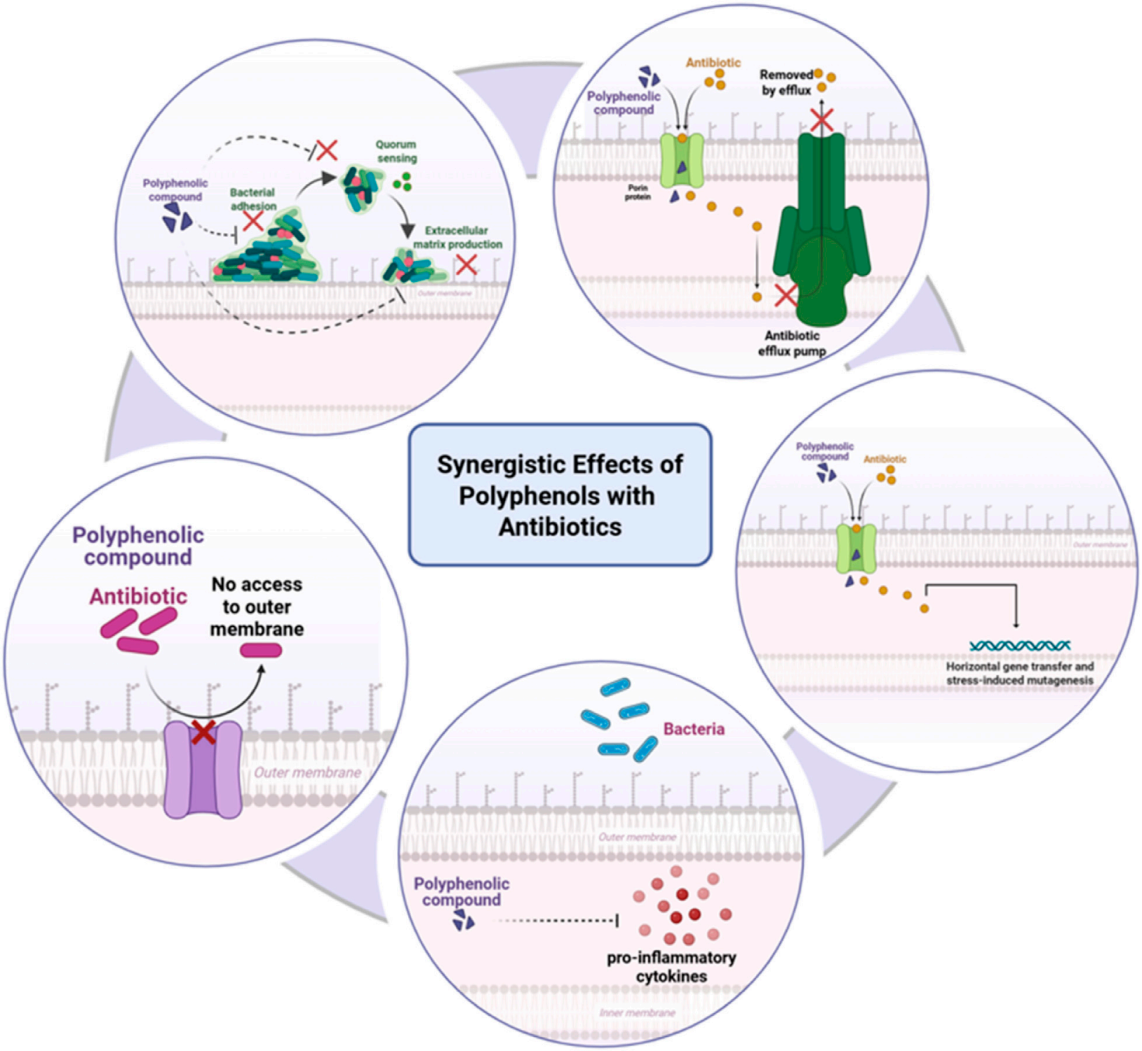
Synergistic effects of polyphenols with antibiotics against multidrug-resistant periodontal pathogens. Polyphenols enhance antibiotic action by disrupting bacterial membranes, inhibiting efflux pumps, interfering with quorum-sensing systems, and suppressing virulence gene expression. They also modulate host inflammatory pathways, reducing pro-inflammatory cytokine release and supporting immune defense. Together, these combined effects potentiate antibiotic efficacy while mitigating resistance. Created with Biorender.com.

Of particular note is the potential for synergistic interaction between polyphenols and antibiotics to be used against resistant strains and to inhibit resistance development (Atta et al., 2023). Sub-inhibitory levels of antibiotics with the addition of polyphenols have been shown to reactivate activity against resistant *A. actinomycetemcomitans* and *P. intermedia* isolates. Polyphenols could also interrupt genetic mechanisms involved in resistance transmission, including horizontal gene transfer and stress-induced mutagenesis. By weakening bacterial virulence without exerting direct lethal force, polyphenols diminish the selective advantage of resistance genes (Ivanov et al., 2022) (Figure 3).

Pharmacologically, these synergistic interactions are avenues for dose-sparing strategies with lowered risks of long-term antibiotic use, such as gastrointestinal dysbiosis and allergic reactions (Kesavelu and Jog, 2023). Moreover, synergy between polyphenols and antibiotics may prolong the useful life of existing antimicrobial compounds while lowering the demand for developing entirely new drugs (De Rossi et al., 2025).

The polyphenol-antibiotic synergism is a scientifically rational and clinically valuable approach to managing multidrug-resistant periodontal infections. With complementary mechanisms of action, targeting bacterial physiology, these combinations enhance antimicrobial action, reduce the risk of resistance, and facilitate healing of host tissues (Kumar et al., 2023). However, to translate these findings into clinical practice, additional studies are needed to fine-tune dosing regimens, delivery (e.g., nanoparticles or local gels), and to demonstrate efficacy in appropriately conducted randomized controlled trials.

### Polyphenols in biofilm disruption and quorum sensing inhibition

Biofilm formation is a hallmark of chronic periodontal disease and a primary cause of antimicrobial resistance and treatment failure (Sharma et al., 2023). In the periodontal pocket, polymicrobial biofilms with intricate architectures, anchored in the subgingival matrix, provide structural and metabolic protection to pathogens such as *P*. *gingivalis*, *A*. *actinomycetemcomitans*, and *T*. *forsythia* (Kuboniwa and Lamont, 2000). These biofilms are not passive havens but rather sophisticated communities governed by quorum sensing (QS) systems—chemical communication systems that regulate virulence, gene expression, and interspecies cooperation (Ng et al., 2016). The ability of polyphenolic compounds to disrupt these systems, both physically and molecularly, positions them as useful non-antibiotic entities in the management of periodontal disease (Li et al., 2025).

Polyphenols exhibit biofilm-disruptive properties through diverse mechanisms (De Rossi et al., 2025). Bacterial adhesion and co-aggregation are early critical events in the development of a biofilm (Sa et al., 2022). Polyphenols such as EGCG, quercetin, and proanthocyanidins have been shown to inhibit bacterial surface adhesins and fimbriae, thus inhibiting their adhesion to epithelial cells and tooth surface (Lombardo et al., 2015). For instance, EGCG strongly downregulates fimA expression in *P. gingivalis* and thereby prevents its primary attachment to gingival crevicular tissue (Li et al., 2024).

In established biofilms, polyphenols may penetrate through the extracellular polymeric substance (EPS) matrix and destabilize the architecture (Fontana et al., 2022). The EPS, composed primarily of polysaccharides, proteins, lipids, and extracellular DNA, is a scaffold that shields bacteria from host immune response as well as antimicrobials. Polyphenols such as curcumin and tannic acid disrupt EPS integrity by chelating metal ions (e.g., calcium and magnesium) that support the biofilm matrix, break down matrix constituents, or induce oxidative stress inhibiting matrix synthesis. Disruption undermines biofilm structure, puts bacteria in contact with environmental insults, and enhances the efficacy of adjunctive therapies (Pinto et al., 2020).

**FIGURE 4:**
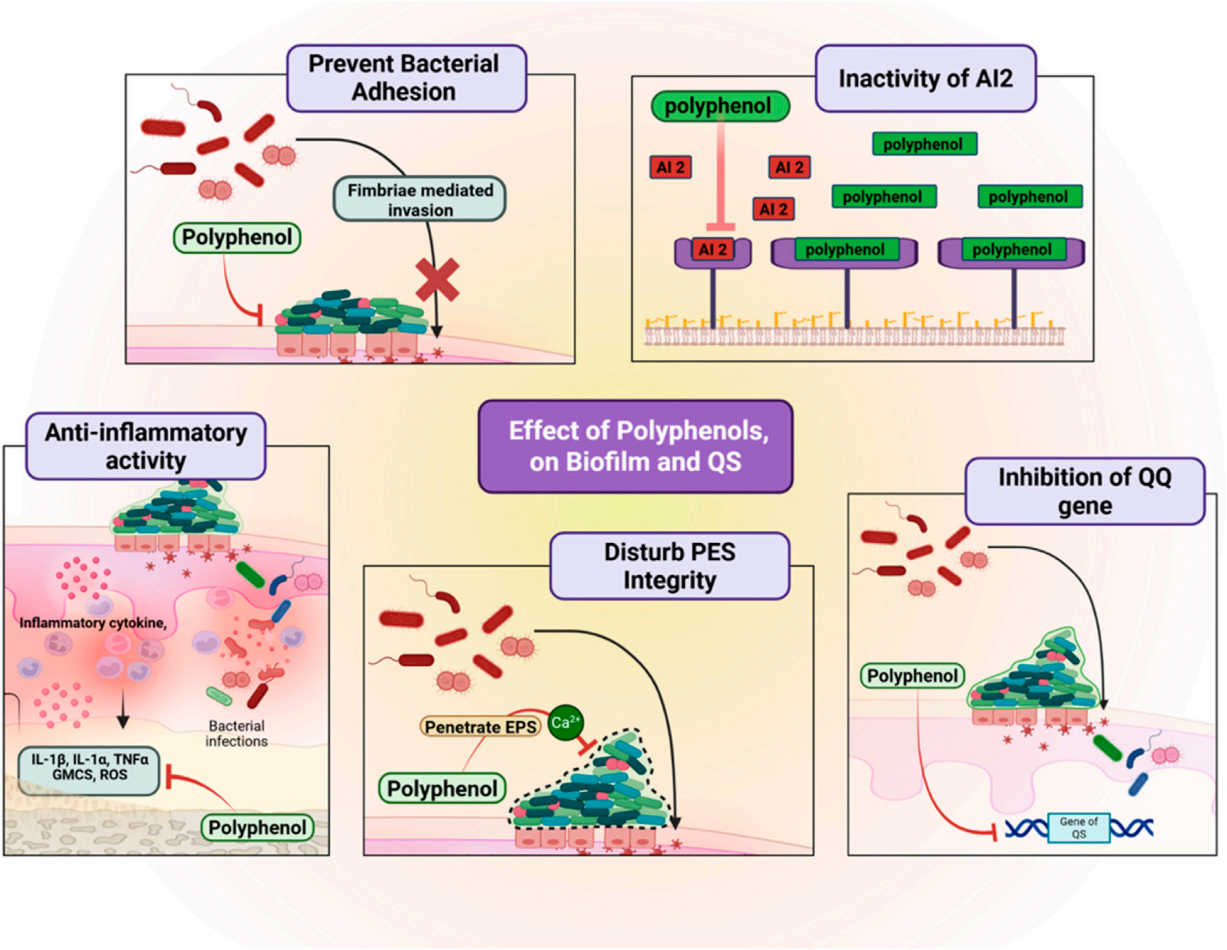
Effects of polyphenols on biofilm formation and quorum sensing in periodontal pathogens. Polyphenols prevent bacterial adhesion by blocking fimbriae-mediated invasion, inactivate autoinducer-2 signaling, inhibit quorum-sensing regulatory genes, and disturb extracellular polymeric substance (EPS) integrity. They also exert anti-inflammatory activity by suppressing NF-κB signaling and reducing pro-inflammatory cytokines (IL-1β, IL-6, TNF-α). These combined actions disrupt biofilm stability and microbial communication, reducing periodontal pathogenicity. Created with Biorender.com.

Besides, polyphenols possess the capacity to inhibit and target QS pathways, which are exploited by bacteria to control concerted actions, including biofilm formation, virulence factor expression, and antibiotic resistance (Lima et al., 2023). QS systems involve the generation and perception of autoinducers, such as acyl-homoserine lactones (AHLs) in Gram-negative bacteria and autoinducing peptides (AIPs) in Gram-positive bacteria. Polyphenols interfere with these systems in a number of different ways: by preventing the synthesis of signaling molecules, by inactivating the autoinducers themselves, or by competitively binding to their receptors (Hetta et al., 2023). For example, naringenin and apigenin inhibit the luxS-dependent AI-2 system in *F. nucleatum*, which is essential for interspecies communication in periodontal biofilms (Ben Lagha et al., 2015). Abating these signal pathways lowers bacterial organization, making the population disordered and vulnerable to immune elimination and antimicrobials (Figure 4).

Polyphenol’s quorum-quenching activity also results in inhibition of significant virulence genes. In *A. actinomycetemcomitans*, for instance, quercetin has been shown to downregulate the leukotoxin gene ltxA, an essential factor for periodontal tissue destruction (Chang et al., 2018). Resveratrol also modulates capsule formation and oxidative stress response-related genes in *P. gingivalis*, which finally suppresses its pathogenicity. This antivirulence strategy—neutralizing rather than killing bacteria—avoids selective pressure for resistance and preserves host–microbe homeostasis (Kugaji et al., 2019).

Interestingly, polyphenols also display potent inhibitory activity against multispecies biofilms, which are more resistant than single-species populations by synergistic defense mechanisms (De Rossi et al., 2025). With co-culture models of *T. forsythia*, *P. gingivalis*, and *F. nucleatum*, studies demonstrated that treatment with extracts rich in polyphenols (e.g., cranberry or green tea) greatly decreased biomass of the biofilm, disrupted interbacterial signaling, and blocked collaborative virulence gene expression (Ben et al., 2017). These findings emphasize the broad-spectrum nature of polyphenol activity and their relevance in real clinical scenarios where periodontal infection is due to mixed microbial flora.

The anti-QS and antibiofilm action of polyphenols is not restricted to *in vitro* conditions. Pilot clinical trials and *in vivo* models have revealed supportive evidence. For example, the topical application of mouthwashes or gels with polyphenols in patients suffering from gingivitis and periodontitis has resulted in reduced plaque indices, reduced bleeding on probing, and improved microbial profiles (Löhr et al., 2015; Granica et al., 2016). These benefits can be attributed not only to direct antibacterial action but also to modulation of biofilm activity and inhibition of inflammatory mediators involved in QS pathways.

Polyphenolic compounds possess a two-pronged mode of action against periodontal pathogens in physically deconstructing biofilms and chemically suppressing quorum sensing networks (Lal et al., 2021). Their multiaction predisposes them optimally to counter the complex microbial ecology of periodontal pockets. Their ability to suppress bacterial coordination, decrease virulence, and enhance susceptibility of pathogens to immune and drug interventions holds much for the future of periodontal therapy. Further clinical trials and formulation efforts are warranted to translate these preclinical and *in vitro* results into successful treatment.

### Safety, cytotoxicity, and host modulation potential of polyphenols

Polyphenols are emerging as promising candidates for adjunctive periodontal therapy on the basis of their potent antimicrobial, anti-inflammatory, and antioxidant properties (Hashim et al., 2024; Hashim et al., 2025a). For clinical use, however, it is necessary to evaluate their safety profiles, cytotoxicity, and modulatory effects on host tissues (Hashim et al., 2024; Hashim et al., 2025a). Unlike many synthetic antimicrobials, polyphenols are relatively nontoxic to mammalian cells at therapeutic concentrations, but their effect is inconsistent on the basis of compound type, concentration, route of administration, and target cell type (Hashim et al., 2025a).

For cytotoxicity and safety, polyphenolic compounds such as EGCG, resveratrol, curcumin, and quercetin have been extensively studied in oral keratinocytes, gingival fibroblasts, and periodontal ligament (PDL) cells (Hattarki et al., 2023). Most of them present a dose-dependent duality: at low concentrations (typically <50 µM), they promote cell growth, collagen synthesis, and wound healing, while at high concentrations (>100 µM), they may induce oxidative stress, mitochondrial dysfunction, or apoptotic pathways (Farag et al., 2025). For example, EGCG at concentrations up to 25 µM is cytoprotective for gingival fibroblasts, while at >100 μM, it is able to reduce viability via increased ROS generation. Optimization of therapeutic windows is, therefore, needed in order to obtain the beneficial effects of polyphenols without modifying cell integrity (Cromie and Gao, 2015).

Importantly, biocompatibility of polyphenols can be significantly enhanced by formulation strategies such as encapsulation in liposomes, nanoparticles, or hydrogel matrices. These delivery systems not only stabilize and enhance bioavailability but also deliver controlled release at target sites while minimizing systemic exposure and cytotoxicity. For instance, curcumin-loaded nanoparticles have shown enhanced periodontal pocket penetration and lowered toxicity to host cells when compared with free curcumin (Pérez-Pacheco et al., 2021).

Apart from safety considerations, polyphenols play a role in host modulation by mitigating the chronic inflammation that is the basis of periodontal tissue destruction. The majority of polyphenols are anti-inflammatory by inhibiting the NF-κB pathway, which is a master regulator of cytokine production (Jayusman et al., 2022). The outcome is the downregulation of pro-inflammatory mediators such as TNF-α, IL-1β, and IL-6 in gingival tissues (Jayusman et al., 2022). Concurrently, polyphenols promote the expression of anti-inflammatory cytokines like IL-10 and modulate macrophage polarization towards the M2 phenotype, which is associated with tissue repair and resolution of inflammation (Jayusman et al., 2022). These effects have been observed both *in vitro* and in experimental models of periodontitis, suggesting that polyphenols can modulate the host immune response towards a regenerative rather than a destructive phenotype (Bunte et al., 2019).

Moreover, polyphenols possess antioxidant activity that resists the oxidative stress of periodontal inflammation. By virtue of their ROS-scavenging ability, upregulation of endogenous antioxidant enzymes (i.e., superoxide dismutase, catalase, glutathione peroxidase), and preservation of mitochondrial function, polyphenols reinstate redox homeostasis in periodontal tissues (Rudrapal et al., 2022). This antioxidant action not only protects gingival cells from oxidative injury but also assists in alveolar bone retention by suppressing osteoclastogenesis and promoting osteoblastic activity.

Additionally, some polyphenols have also demonstrated potential in modulating tissue remodeling and regeneration. Quercetin, for instance, induces type I collagen synthesis and enhances the expression of growth factors such as TGF-β1 and VEGF in gingival fibroblasts, which can heal wounds and induce angiogenesis (Mi et al., 2022). Resveratrol has been shown to induce osteogenic differentiation of PDL stem cells through activation of the SIRT1/Runx2 pathway, further enhancing its application in periodontal regeneration (Wang et al., 2018).

Cytocompatibility and safety of polyphenols are favorable in optimal dosing regimens, and their immune and oxidative response modulating activity further improves the therapeutic profile. The molecules not only inactivate pathogens but also cure tissue, regulate inflammation, and preserve periodontal architecture and are hence ideal for integrative approaches in periodontal management. Future research must be focused on the optimization of delivery systems, long-term *in vivo* studies, and confirmation of host-modulatory activities in clinical trials to ascertain their safety and efficacy in humans (Rudrapal et al., 2022; Wang et al., 2018).

### *In vitro* evidence of polyphenol efficacy against MDR periodontal bacteria

Numerous *in vitro* studies have shown that polyphenolic compounds are very effective at fighting bacteria that are resistant to multiple drugs, including *P*. *gingivalis*, *A*. *actinomycetemcomitans*, *T*.*forsythia*, and *F*. *nucleatum* (Izui et al., 2015; Kumbar et al., 2021). These studies consistently show that polyphenols like EGCG, quercetin, curcumin, and resveratrol have minimum inhibitory concentrations (MICs) that are effective in real-world situations, often similar to or better than standard antibiotics against multidrug-resistant strains (Jayusman et al., 2022; Izui et al., 2015; Kumbar et al., 2021). Polyphenols work in several ways—breaking down bacterial membranes, preventing biofilm formation, and blocking communication between bacteria—unlike traditional antimicrobials that usually target just one pathway (Makarewicz et al., 2021). Importantly, even in bacteria that are already resistant to antibiotics like metronidazole, doxycycline, or amoxicillin, polyphenols have been shown to significantly reduce their growth, indicating that they can work around the usual resistance methods. These findings provide a strong rationale for advancing polyphenols into clinical formulations, especially for patients with refractory or recurrent periodontal infections (Cione et al., 2019; Mancuso et al., 2021).

For a comparative overview of current therapeutic modalities, their limitations, and examples of non-polyphenolic phytochemicals with reported anti-periodontitic activity, readers are referred to Supplementary Table S1.

### Challenges in bioavailability and delivery of polyphenolic agents

**FIGURE 5:**
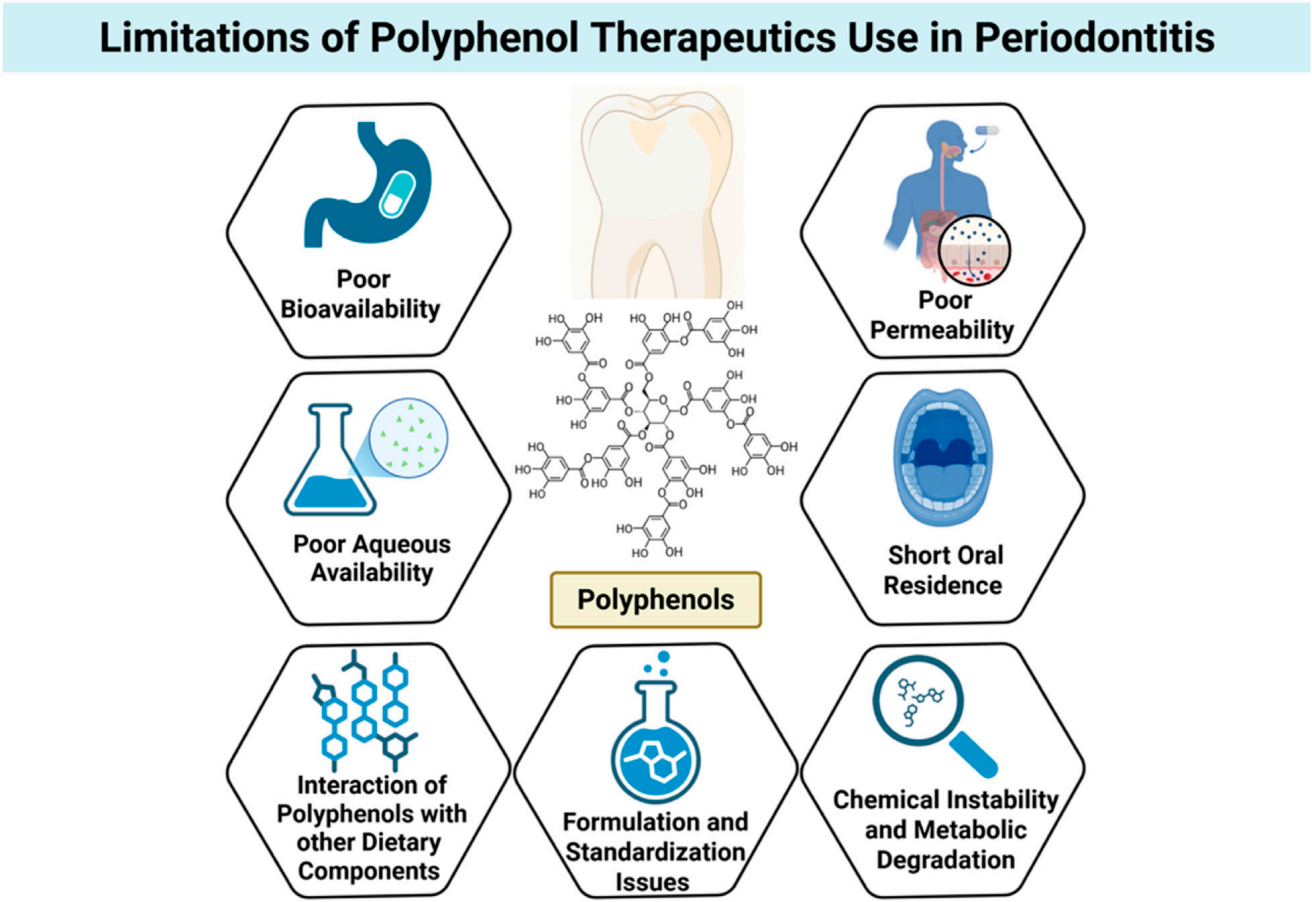
Limitations of polyphenol use in periodontal therapeutics. Despite their promising antimicrobial and host-modulatory effects, polyphenols face several barriers to clinical application, including poor bioavailability, low aqueous solubility, limited permeability, short oral residence time, and susceptibility to chemical instability and metabolic degradation. Additional challenges include interactions with dietary components and inconsistencies in formulation and standardization, all of which hinder translation into effective periodontal therapies. Created with Biorender.com.

While polyphenolic compounds display impressive antimicrobial, antioxidant, and host-modulatory activities *in vitro*, their clinical utility is frequently undermined by poor bioavailability and delivery issues. Such limitations dramatically restrict their therapeutic usefulness, particularly for treating chronic oral diseases such as periodontitis, where site-directed activity, controlled release, and local bioactivity are essential for clinical success (Kumbar et al., 2025; He et al., 2020; Zheng et al., 2024; Ofoezie et al., 2025) (Figure 5).

One of the most notable difficulties is the low aqueous solubility of many polyphenols, particularly flavonoids like quercetin, apigenin, and curcumin. Hydrophobicity hinders dissolution in physiological fluids, reducing absorption across biological membranes. Even polyphenols of relatively higher polarity, like catechins, face solubility issues upon preparation in aqueous oral rinses or gels. Thus, a major portion of orally or topically administered polyphenols fails to reach therapeutic concentrations at the target site (Alizadeh et al., 2023).

Aside from solubility issues, polyphenols are also very susceptible to chemical instability and metabolic degradation due to several factors. Many easily oxidize when exposed to light, heat, or pH changes, which is particularly relevant in the oral cavity where there are temperature gradients, enzyme activity, and microbial byproducts (Zagoskina et al., 2023). Polyphenols also face extensive first-pass metabolism when administered systemically, further contributing to their suboptimal bioavailability. Enzymatic conjugation via glucuronidation, sulfation, and methylation in the liver and intestinal wall considerably reduces the bioactive free polyphenol forms that can exert pharmacological activity (Zagoskina et al., 2023).

The second big obstacle is their poor permeability through the mucosa and short residence time in the oral cavity. Salivary flow, mechanical clearance, and swallowing account for the rapid removal of locally administered agents from gingival or mucosal surfaces (Harris and Robinson, 1992). This is especially troublesome in periodontal therapy, where the long-term retention of the bioactive agent within the periodontal pocket is extremely beneficial for therapeutic effect. Most conventional forms, i.e., mouthwashes or gels, fail to impart sufficient substantivity to maintain therapeutic levels over time (Bartlett and Maarschalk, 2012).

Further complicating delivery is the interaction of polyphenols with other dietary components or biomolecules, which can impact absorption, stability, and activity. Polyphenols are prone to non-specific binding to proteins, lipids, and metal ions, forming complexes that may be biologically inactive or poorly absorbed. These interactions can significantly reduce the free fraction available for interaction with microbial targets or host cells (Oliveira et al., 2025).

From a formulation standpoint, polyphenols are difficult in that they possess heterogeneous molecular weights and chemical properties, and standardization is difficult. Natural product extracts are often complex mixtures of polyphenols of undefined bioactivity and variable pharmacokinetics. Batch-to-batch variability, low purity, and irregular loading into delivery vehicles render their utilization in the clinic problematic and regulatory approval difficult (Stromsnes et al., 2021).

To surmount these challenges, research has increasingly been conducted on the application of new drug delivery systems, including nanoparticles, liposomes, mucoadhesive patches, and *in situ* gelling systems. These systems aim to stabilize the polyphenols against degradation, increase their solubility, facilitate tissue penetration, and provide controlled release. These technologies are, however, also connected with limitations such as complexity in fabrication, potential toxicity of the carrier, stability during storage, and scalability for clinical use (Ezike et al., 2023).

While polyphenols have tremendous potential for the treatment of periodontal disease, their current clinical usefulness is hindered by several pharmacokinetic and formulation-related drawbacks. Overcoming constraints related to solubility, stability, mucosal retention, and targeted delivery remains important in the pursuit of polyphenol-based therapeutics. Advances in formulation science and delivery technology will be critical to the full exploitation of these naturally occurring compounds for therapeutic advantage in periodontal as well as general oral healthcare (Stromsnes et al., 2021; Ezike et al., 2023).

### Nanoformulations and drug delivery systems for polyphenol enhancement

**FIGURE 6:**
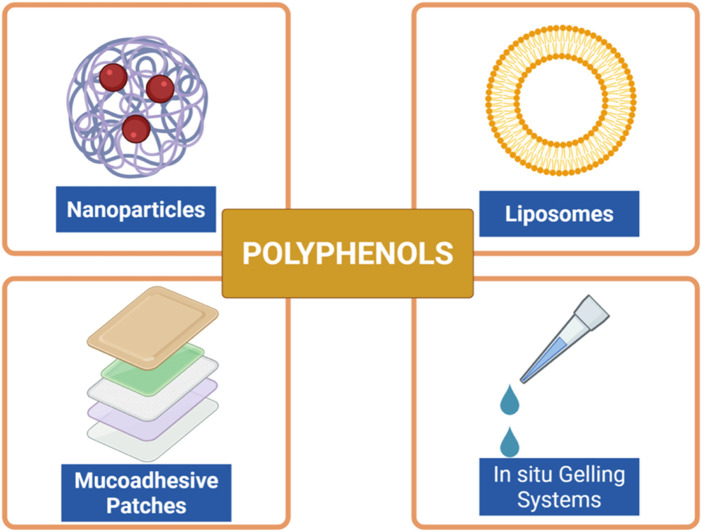
Advanced delivery systems for polyphenols in periodontal therapy. Various strategies have been developed to improve solubility, stability, bioavailability, and site-specific delivery of polyphenolic compounds. Nanoparticles and liposomes enhance cellular uptake and protect against degradation, mucoadhesive patches prolong residence time in the oral cavity, and *in situ* gelling systems allow controlled and sustained local release. These novel formulations aim to overcome pharmacokinetic limitations and optimize the therapeutic potential of polyphenols in periodontitis. Created with Biorender.com.

**FIGURE 7:**
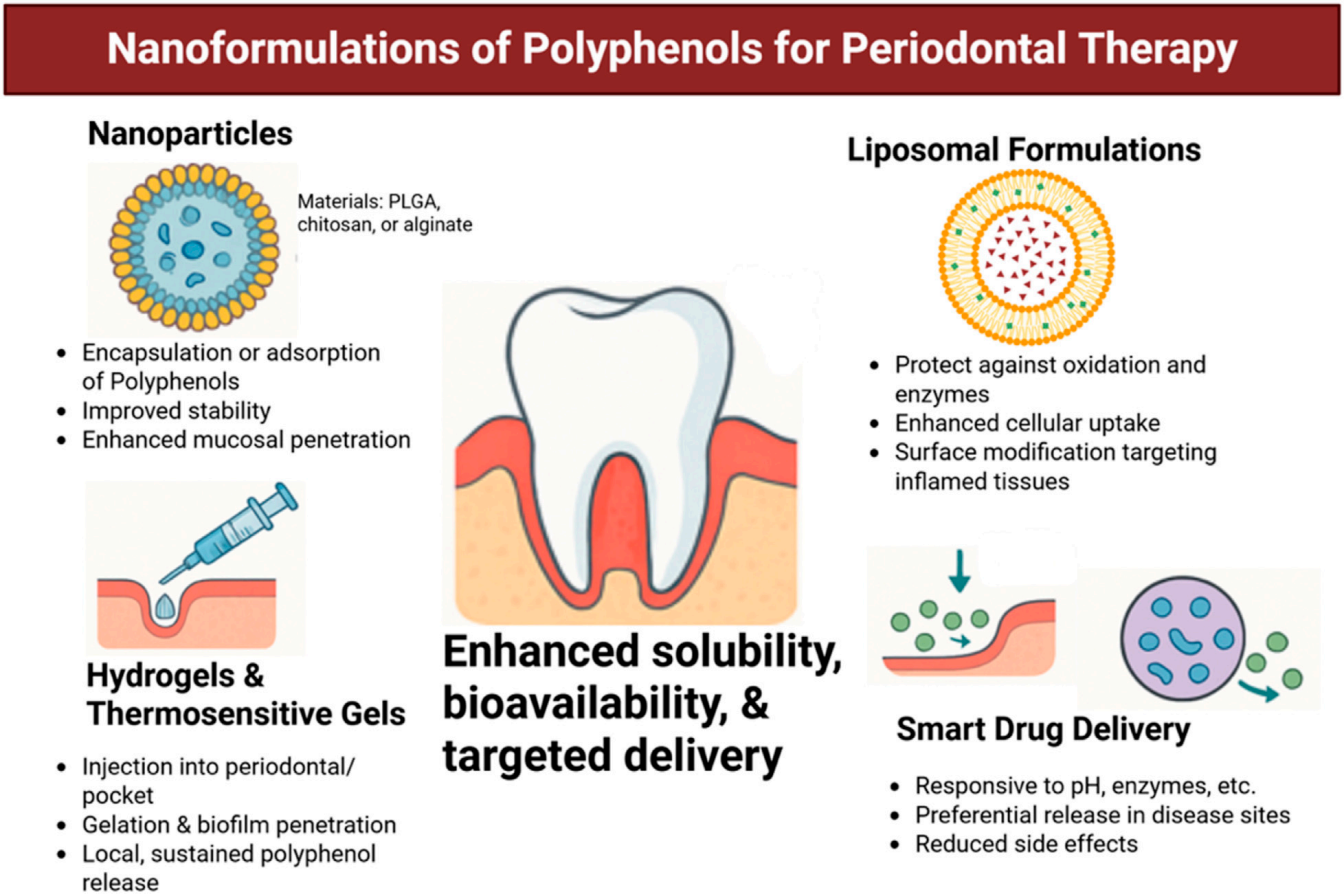
Nanotechnology-based delivery systems of polyphenols for periodontal therapy. Various formulations have been developed to overcome poor solubility, instability, and low bioavailability of polyphenols. Nanoparticles (e.g., PLGA, chitosan, alginate) enable encapsulation and enhanced mucosal penetration; liposomal formulations protect against enzymatic degradation and facilitate uptake into inflamed tissues; hydrogels and thermosensitive gels provide local, sustained release within periodontal pockets; and smart drug delivery platforms allow stimuli-responsive release, reducing systemic side effects. Together, these approaches improve therapeutic precision and efficacy of polyphenols in periodontal treatment. Created with Biorender.com.

Even though polyphenols have great potential for treating periodontal disease, their clinical application is greatly restricted due to problems like not dissolving well in water, breaking down easily in the body, being quickly processed, and not being absorbed well (Basu et al., 2018). Numerous studies are looking into using nanoformulations and advanced drug delivery methods to address these challenges and enhance the effectiveness of polyphenolic compounds in the body (Martinho et al., 2011; Arayne et al., 2007; Mirza and Siddiqui, 2014). These delivery platforms improve stability and site-specific accumulation and enable controlled and sustained release, offering significant potential benefits for localized periodontal therapy (Basu et al., 2018; Martinho et al., 2011) (Figures 6, 7). Among the most widely studied systems are nanoparticles typically composed of biodegradable polymers such as poly (lactic-co-glycolic acid) (PLGA), chitosan, or alginate. These nanoparticles encapsulate polyphenols within their matrix or adsorb them onto their surface, protecting them from degradation and allowing for efficient mucosal penetration (Akdaşçi et al., 2024). For example, PLGA nanoparticles filled with curcumin showed a better ability to fight bacteria like *P. gingivalis* and *A. actinomycetemcomitans* in lab tests, and they were also safer for use and stayed longer in the gum area (Pan et al., 2020). Similarly, EGCG-encapsulated chitosan nanoparticles retained antibacterial efficacy and exhibited superior adhesion to mucosal tissues, making them ideal for topical application (Loo et al., 2022). Liposomal formulations, which are tiny round structures made of layers of phospholipids, have also been used to deliver polyphenols like quercetin and resveratrol (Nsaira et al., 2022). Liposomes are safe to use in the body and can hold both water-soluble and fat-soluble substances, protecting them from damage by oxidation and enzymes. Their fluidic membranes help them merge with bacterial cell walls or gum tissue, making it easier for cells to take them in and boosting their ability to fight germs. Additionally, changing the surface with substances like hyaluronic acid or antibodies can help these particles better target areas in the gums that are inflamed or infected (Dejeu et al., 2024).

Solid lipid nanoparticles (SLNs) and nanostructured lipid carriers (NLCs) represent another promising class of delivery systems for polyphenols (Nsaira et al., 2022). These lipid-based carriers take the best features of liposomes and polymeric nanoparticles, providing great stability, compatibility with living tissues, and controlled release of substances. NLCs filled with curcumin or green tea catechins have shown not only stronger ability to fight germs but also greater anti-inflammatory benefits when used on swollen gingival tissues in early research models (Tang et al., 2022).

Besides nanoparticles, hydrogels and temperature-sensitive gels have been created to deliver treatments directly to the gums. These systems can be injected straight into the periodontal pocket, where they harden when they come into contact with body temperature or certain salts, gradually releasing polyphenols. Hydrogels incorporating polyphenol-loaded nanoparticles further enhance local retention and penetration into biofilms (Wang et al., 2023). For instance, a system that delivers both doxycycline and nanoparticles filled with resveratrol, placed in a chitosan hydrogel, has been effective in lowering biofilm size and inflammation in models of gum disease (Li et al., 2022).

Another innovative strategy involves mucoadhesive films and nanofibers, which can be applied directly to gingival surfaces or within periodontal pockets (Zhan and Löwik, 2019). These systems allow prolonged contact with the target site and provide protection from salivary dilution. Electrospun nanofibers that include polyphenols like tannic acid or ellagic acid have shown they can kill germs and help skin cells grow and produce collagen, which supports the healing of gums (Momose et al., 2016).

Nanocarriers can be tailored to respond to environmental stimuli such as pH, temperature, or enzymatic activity. In the inflamed area of the gums, which has a low pH and high levels of matrix metalloproteinases (MMPs), these responsive systems assure that drugs are released only where needed, reducing side effects and improving treatment effectiveness (Naveedunissa et al., 2025). pH-sensitive liposomes, for example, can release their polyphenol payload preferentially in acidic periodontal pockets, thereby enhancing local drug concentration while sparing healthy tissues (Taneja et al., 2025).

Nanoformulations and advanced drug delivery systems are a groundbreaking way to enhance the effectiveness of polyphenolic compounds in treating gum disease. By improving solubility, bioavailability, and targeted delivery, these technologies overcome the limitations of conventional polyphenol administration. The integration of nanotechnology with polyphenol-based therapeutics holds the potential to completely transform the management of periodontal disease by offering localized, efficient, and minimally invasive treatment options.

### Polyphenols versus traditional antimicrobials

While the classical antimicrobials are known to exert well-documented bactericidal or bacteriostatic activity, their prolonged administration is commonly associated with the drawbacks of microbial resistance, tolerance to biofilm, dysbiosis, and host tissue side effects (Salam et al., 2022). Polyphenols, in contrast, act via multi-targeted action—interference with biofilms, quorum sensing inhibition, reduced inflammation, and enhanced host defense—without exerting selective pressure leading to resistance (Mirghani et al., 2022). Comparative evidence suggests that while polyphenols may be less effective in eradication of fast-growing bacteria, they are more favorable in biocompatibility, broader action spectra, and longer-term sustainability of controlling chronic periodontal infections (Higuchi et al., 2024; Guo et al., 2022).

A detailed comparison of polyphenols and conventional antimicrobials in periodontal therapy, covering mechanisms of action, resistance development, biofilm activity, host modulation, and formulation challenges, is presented in Supplementary Table S2.

### Clinical potential and translational gaps in periodontal therapy

Despite their antimicrobial, anti-inflammatory, antioxidant, and host-modulatory benefits, polyphenolic compounds such as EGCG, resveratrol, curcumin, and quercetin, though effective in inhibiting periodontal pathogens, disrupting biofilms, and promoting tissue regeneration in preclinical models, still face significant translational challenges (Hashim et al., 2025b; Jayusman et al., 2022). The primary limitation lies in the lack of large-scale, standardized randomized controlled trials assessing their efficacy, safety, optimal dosing, and delivery in human periodontitis. Most existing clinical studies are small-scale, short-term, and inconsistent in design and formulation, hindering the ability to generalize findings or establish clinical guidelines (Hashim et al., 2025b; Jayusman et al., 2022).

Yet another significant barrier is the uncontrolled bioavailability and pharmacokinetics of polyphenols on systemic or topical administration. Individual-to-individual variations in metabolism, absorption, and clearance complicate attempts at optimizing dosing and reproducing therapeutic outcomes. Moreover, the preparations used in medical settings—e.g., mouthwashes, gels, or supplements—generally are not drug-delivery technology advanced enough to offer sustained release, site-specific delivery, or resistance to enzymatic degradation in the oral environment (Rubió et al., 2014).

Commercial and regulatory factors also contribute to the translational bottleneck. Unlike synthetic drugs, polyphenols are typically considered nutraceuticals or herbal products, and this leads to irregular regulatory control, minimal financial investment, and inability to protect against intellectual property theft. Polyphenol variation in source, purity, and extraction process leads to variation in active ingredient composition, which also makes it challenging to standardize and control dosages (Nasri et al., 2014).

From the clinical integration perspective, implementation of polyphenols into everyday periodontal therapy should be accompanied not only by proof of efficacy but also by safety evaluation, cost-effectiveness, and compatibility with established treatment approaches. Therefore, e.g., nothing is known about whether polyphenol-containing treatments can replace or more likely supplement scaling and root planing, locally used antimicrobials, or systemic antibiotics. Additionally, long-term effects of recurrent polyphenol exposure on oral microbiota and host tissues remain poorly explored. To bridge these translational deficits, interdisciplinary research is needed. Interdisciplinary cooperation between periodontists, pharmacologists, formulation scientists, and regulatory scientists will be needed to develop standard, stable, and bioavailable polyphenol delivery systems tailored for the periodontal pocket. At the same time, properly designed clinical trials with robust methodologies, adequate sample sizes, and standardized outcome variables must be given high priority in order to develop high-level evidence. Further, real-world patient compliance, patient satisfaction, and outcome collection data will become relevant to support clinical guidelines and regulatory approval.

The potential for clinical application of polyphenols in periodontal treatment is underpinned by an increasingly robust preclinical and mechanistic evidence base. However, a range of translational hurdles—ranging from formulation limitations and regulatory ambiguity to the lack of high-quality clinical evidence—must be overcome before it becomes possible to introduce these medicines into routine periodontal therapy with confidence. By solving each of these issues sequentially, polyphenol-based therapies may shortly become evidence-based, useful adjuncts in the treatment of periodontal disease.

## Future directions and emerging polyphenol-based therapeutics

As the burden of antimicrobial resistance expands, and conventional treatments for periodontal disease become increasingly throttled, polyphenol-based medications are emerging as an exponentially thrilling Frontier. The future research horizons for this area are poised to steer the focus away from mere antmicrobial activity to multifunctional, host-modulatory, and precision-guided therapies. One of the most promising fields is the development of smart delivery systems—pH-responsive, enzyme-sensitive, and biofilm-permeating nanocarriers—that can offer site-specific, controlled release of polyphenols in periodontal pockets. These delivery systems are meant to deliver therapeutic levels of polyphenols over extended periods of time, minimize systemic exposure, and avoid the fast breakdown and clearance processes presently limiting clinical efficacy. Another emerging strategy is combination therapeutics, in which polyphenols are co-delivered with conventional antibiotics, anti-inflammatory agents, or regenerative biomolecules. The combinations have the potential to synergize to enhance microbial eradication, host immune response modulation, and tissue healing. Dual-delivery systems of resveratrol and doxycycline or EGCG and statins are being investigated for their potential to target pathogenic bacteria simultaneously and inhibit osteoclastic activity, thus offering both infection control and bone preservation.

In parallel, omics-based and systems biology approaches—transcriptomics, proteomics, and metabolomics—are being investigated to unravel complex molecular interactions of polyphenols with the oral microbiome and host immune systems. These platforms may be used for the discovery of response biomarkers for therapy, modeling synergistic relationships, and tailoring treatment modalities based on microbial or genetic signatures. This promises precision polyphenol therapy, where interventions are tailored to specific patients based on microbial status, inflammatory status, and genetic risk of periodontitis.

Lastly, an expanded therapeutic perspective considers the effect of polyphenols not just as localized antimicrobials but also as oral-systemic health integration compounds. In view of the systemic inflammatory connections that have been postulated between periodontitis and conditions such as diabetes, cardiovascular disease, and Alzheimer’s disease, polyphenols can be explored in the future as bi-functional compounds imparting benefits on both periodontal tissues as well as overall health. Their antioxidant, immunomodulatory, and endothelial-protective activities position them ideally for this.

The future of polyphenol therapeutics in periodontal medicine is both exciting and expansive. With advances in formulation technology, synthetic chemistry, systems biology, and clinical integration, polyphenols would likely emerge from experimental adjuncts to become mainstay therapeutic agents—safe, effective, and sustainable treatments for the management of complex periodontal infections and beyond.

## Conclusion and implications for periodontal disease management

The growing issue of antimicrobial resistance, as well as the limitations of conventional periodontal therapies, necessitates the emergence of safe, effective, and multi-targeted therapy strategies. Polyphenolic phytochemicals, derived from an exceedingly diverse array of plant sources, have emerged as highly promising agents in this regard owing to their broad-spectrum antimicrobial, anti-biofilm, quorum-sensing inhibitory, antioxidant, and host-modulatory properties. From *in vitro* systems to preclinical animal models, polyphenols have consistently proven themselves capable of inhibiting key periodontal pathogens, disrupting pathogenic biofilms, inhibiting inflammatory responses, and stimulating tissue repair and regeneration. Advances in drug delivery—notably nanoformulations and mucoadhesive systems—are progressively addressing fundamental shortcomings such as limited solubility, poor bioavailability, and brief retention in the periodontal environment. Nonetheless, because of their desirable pharmacological profile, the incorporation of polyphenols into standard periodontal treatment is just beginning. Translational shortcomings such as formulation heterogeneity, regulatory uncertainty, and a lack of standard, large-scale clinical trials need to be closed to establish efficacy and assure safety in practice. All such future advancements should be directed toward precision-targeted formulations, synergistic combinations with presently available antimicrobials or regenerative agents, and microbiome and immune profiling-based individualized treatments.

Finally, polyphenol therapeutics hold deep significance for the treatment of periodontal disease. The agents not only offer a non-antibiotic method of microbial suppression but also a method of host modulation such that healing and homeostasis are encouraged. Their potential for augmenting oral as well as systemic health renders them of particular interest for holistic and preventive models of dental treatment. With ongoing scientific and clinical advancements, polyphenols may soon transition from promising bioactives to essential components of integrative periodontal therapy.
