# Supplementary material for: Polyphenolic compounds in combating MDR periodontal pathogens: current research and future directions
Source: Front Pharmacol. 2025 Nov 12;16:1678979. doi: 10.3389/fphar.2025.1678979 (PMC12647076; doi:10.3389/fphar.2025.1678979)
Supplement: Supplementary file 1 [file Table1.docx]

**Supplementary Table 1.** Current therapeutic strategies for periodontitis, their mechanisms of action, associated limitations, and examples of phytochemicals other than polyphenols with reported anti-periodontitic activity. The table highlights conventional antibiotics, adjunctive agents, and phytochemical classes such as alkaloids, terpenoids, saponins, and polysaccharides. This overview provides a broader therapeutic context, positioning polyphenols as part of an integrative approach to combating antimicrobial resistance and biofilm-associated periodontal disease.

| Treatment / Agent | Mechanism of Action | Limitations / Side Effects | Examples of Non-Polyphenolic Phytochemicals with Anti-Periodontitic Activity |
| --- | --- | --- | --- |
| Conventional antibiotics (e.g., amoxicillin, metronidazole, doxycycline) | Inhibit bacterial growth, disrupt protein/DNA synthesis | Antimicrobial resistance, gastrointestinal upset, allergic reactions, microbiome dysbiosis | — |
| **Antimicrobial mouthrinses** (e.g., chlorhexidine, essential oils) | Disrupt bacterial membranes, reduce plaque and gingivitis | Tooth staining, taste alteration, mucosal irritation, limited long-term compliance | — |
| **Host-modulating agents** (e.g., subantimicrobial doxycycline, NSAIDs, bisphosphonates) | Inhibit matrix metalloproteinases (MMPs), reduce inflammation, modulate bone resorption | Gastrointestinal irritation, cardiovascular risks, antibiotic resistance (with doxycycline), osteonecrosis risk | — |
| **Laser/photodynamic therapy** | Generate reactive oxygen species, destroy bacterial cells, reduce inflammation | Costly equipment, operator variability, transient post-op sensitivity | — |
| **Probiotics / prebiotics** | Restore microbial balance, compete with pathogens, modulate immune response | Strain-specific efficacy, variable colonization, short-term benefits | *Lactobacillus reuteri*, *Bifidobacterium longum* |
| **Alkaloids** | Antimicrobial, anti-inflammatory, disrupt bacterial cell wall | Limited clinical studies, potential toxicity at high doses | Berberine, matrine |
| **Terpenoids** | Antimicrobial, anti-inflammatory, quorum-sensing inhibition | Low solubility, variable bioavailability | Carvacrol, thymol, eugenol |
| **Saponins** | Membrane disruption, immune-modulatory activity | Hemolytic potential, limited stability | Ginsenosides, glycyrrhizin |
| **Polysaccharides** | Biofilm disruption, immune modulation | Limited mechanistic studies, need for standardization | Aloe vera polysaccharides, chitosan |

**Supplementary Table 2.** *This table provides a comparative overview of polyphenols and traditional antimicrobials in periodontal therapy. It highlights differences in mechanisms of action, biofilm disruption, resistance development, host modulation, cytotoxicity, systemic side effects, formulation challenges, and clinical applicability. While traditional antimicrobials remain widely used, their effectiveness is increasingly undermined by multidrug resistance, whereas polyphenols offer a multi-targeted, biocompatible alternative, though translation to clinical use remains limited by formulation and bioavailability challenges.*

| Parameter | Polyphenols | Traditional Antimicrobials |
| --- | --- | --- |
| Mechanism of Action | Multi-targeted: membrane disruption, quorum sensing inhibition, efflux pump modulation, anti-inflammatory pathways | Target-specific: inhibition of protein synthesis, DNA replication, or cell wall synthesis |
| **Biofilm Disruption** | Inhibit adhesion, prevent maturation, and promote biofilm dispersion | Limited penetration into mature biofilms; reduced efficacy once EPS matrix is established |
| **Resistance Development** | Low tendency; act on multiple targets, reducing selective pressure | High; frequent emergence of multidrug resistance via efflux pumps, enzymatic degradation, and target-site mutations |
| **Quorum Sensing Interference** | Effective; downregulate signaling pathways and virulence gene expression | Rarely targeted |
| **Host Modulation** | Anti-inflammatory, antioxidant, immunomodulatory effects | Minimal host-modulatory effects; risk of dysbiosis due to microbiome disruption |
| **Tissue Cytotoxicity** | Low at optimal concentrations; good biocompatibility with fibroblasts/keratinocytes | Potential cytotoxicity to host tissues at therapeutic doses |
| **Systemic Side Effects** | Generally minimal when applied locally; oral/topical use considered safe | Gastrointestinal upset, allergic reactions, microbiome imbalance, superinfections |
| **Formulation Challenges** | Poor solubility, instability, low bioavailability; overcome with nanocarriers, liposomes, mucoadhesive systems | Widely available in stable, standardized formulations |
| **Spectrum of Activity** | Broad; effective against multiple oral pathogens and inflammatory pathways | Narrow to broad, depending on class; prone to resistance |
| **Sustainability** | Derived from renewable natural sources; potential eco-friendly therapeutic option | Synthetic; long-term use contributes to AMR burden and environmental contamination |
| **Clinical Evidence** | Limited to preclinical and small-scale studies; few robust clinical trials | Well-established evidence base, though efficacy declining due to resistance |
